# A Comprehensive Evaluation of the Impact of Bovine Milk Containing Different Beta-Casein Profiles on Gut Health of Ageing Mice

**DOI:** 10.3390/nu12072147

**Published:** 2020-07-19

**Authors:** Barbara Guantario, Marzia Giribaldi, Chiara Devirgiliis, Alberto Finamore, Elena Colombino, Maria Teresa Capucchio, Rocchina Evangelista, Vincenzo Motta, Paola Zinno, Simona Cirrincione, Sara Antoniazzi, Laura Cavallarin, Marianna Roselli

**Affiliations:** 1 Research Centre for Food and Nutrition, CREA (Council for Agricultural Research and Economics), Via Ardeatina 546, 00178 Rome, Italy; barbara.guantario@crea.gov.it (B.G.); chiara.devirgiliis@crea.gov.it (C.D.); alberto.finamore@crea.gov.it (A.F.); paola.zinno@crea.gov.it (P.Z.); marianna.roselli@crea.gov.it (M.R.); 2 Research Centre for Engineering and Agro-Food Processing, CREA (Council for Agricultural Research and Economics), Strada delle Cacce 73, 10135 Turin, Italy; marzia.giribaldi@crea.gov.it; 3 Department of Veterinary Sciences, University of Turin, Largo Braccini 2, 10095 Grugliasco (TO), Italy; elena.colombino@edu.unito.it (E.C.); mariateresa.capucchio@unito.it (M.T.C.); rocchinae@hotmail.it (R.E.); 4 Institute of Sciences of Food Production, CNR, Largo Braccini 2, 10095 Grugliasco (TO), Italy; simona.cirrincione@ispa.cnr.it (S.C.); sara.antoniazzi@ispa.cnr.it (S.A.); 5 Department of Translational Research on New Technologies in Medicine and Surgery, University of Pisa, Via Savi 10, 56126 Pisa, Italy; vincenzo.motta@med.unipi.it

**Keywords:** A2 beta-casein, elderly, immunosenescence, gut morphology, gut microbiota, gut inflammation, SCFAs

## Abstract

Ageing is often characterised by nutritional deficiencies and functional alterations of the digestive and immune system. The aim of the present study was to analyse the impact of consumption of conventional milk with A1/A2 beta-casein, compared to milk containing only the A2 beta-casein variant, characterised by a protein profile favouring gut health. Twenty-four ageing Balb-c mice (20 months old) were fed for 4 weeks, with either a control diet (CTRL), a diet supplemented with bovine milk containing A1/A2 beta-casein (A1A2) or a diet containing A2/A2 beta-casein (A2A2). Lymphocyte subpopulations, enzymatic activities, cytokine secretion, gut morphology and histopathological alterations were measured in different gut segments, while short-chain fatty acids (SCFAs) content and microbiota composition were evaluated in faecal samples. The A2A2 group showed higher content of faecal SCFAs (in particular, isobutyrate) of intestinal CD4^+^ and CD19^+^ lymphocytes in the intraepithelial compartment and improved villi tropism. The A1A2 group showed higher percentages of intestinal TCRγδ^+^ lymphocytes. Faecal microbiota identified *Deferribacteriaceae* and *Desulfovibrionaceae* as the most discriminant families for the A2A2 group, while *Ruminococcaceae* were associated to the A1A2 group. Taken together, these results suggest a positive role of milk, in particular when containing exclusively A2 beta-casein, on gut immunology and morphology of an ageing mice model.

## 1. Introduction

Milk is an important component of the diet and a source of lipids, carbohydrates (mainly lactose), proteins with high biological value, minerals, like calcium, phosphorous, magnesium, several trace elements, like zinc and iodine, as well as B2, B12, D and A vitamins [1]. Cow’s milk contains about 32 g protein per litre, 80% of which are caseins and 20% whey proteins. Beta-casein, a 209-amino acid protein, is the second most abundant casein in bovine milk and represents about 30% of total caseins. Several genetic variants of beta-casein have been described (UniProtKB, accession number P02666), among which the most represented are named as A1 and A2. The amount of A1 and A2 beta-casein variants in milk depends on the breed of cattle: African and Asian species produce milk containing only the A2 variant, while European cattle produce mainly A1 beta-casein. The milk commercially produced in many countries contains a mixture of both variants, in different proportions [2].

The A1 and A2 beta-casein variants differ for a point mutation in the amino acid sequence at position 67, due to a single C > A substitution in the corresponding gene: the protein sequence of the A1 variant results in a Histidine (His^67^), whereas the A2 form has a Proline (Pro^67^). This difference in amino acid sequence seems to impact on gastrointestinal digestion of beta-casein, as the presence of His^67^ renders the protein susceptible to proteolytic cleavage by digestive enzymes, producing the release of one short beta-casomorphin (BCM) peptide, named BCM-7 [3]. BCM peptides are μ-opioid receptor ligands that include different forms, such as BCM-5, BCM-7 and BCM-9. Among these peptides, BCM-7 has been widely studied in human medicine as it seems to be implicated in a wide range of clinical disorders, including abnormal gastrointestinal function [4], cardiovascular diseases [5], type 1 diabetes [6], schizophrenia and autism [7]. In 2009, a scientific opinion from the European Food Safety Agency (EFSA) concluded that there was no sufficient scientific evidence to require a formal risk assessment for beta-casomorphins and related peptides on human health, due to the absence of a clear cause–effect relationship between the oral intake of BCM-7 or related peptides and aetiology or course of any suggested non-communicable diseases. EFSA opinion, on the other hand, agreed that casomorphins can exert different actions in the intestinal lumen and mucosa, including regulatory effects on gastrointestinal motility and gastric and pancreatic secretions [8].

More recently, further investigations in animal models and in humans have focused on the effects of A1 and A2 beta-casein on the gastrointestinal tract, and on BCM-7 involvement on the intestinal activity [3]. In a rat model, Barnett et al. [9] demonstrated that consumption of milk containing A1 beta-casein caused a delay of gastrointestinal transit time through an opioid-mediated effect and an increased activity of jejunal dipeptidyl peptidase (DPP)-4, a digestive enzyme expressed on brush border membrane and capable of catabolising BCM-7. Moreover, the activity of the inflammatory marker myeloperoxidase (MPO) was increased in the colon. Also, in the murine gut, A1 beta-casein induced an inflammatory response with an upregulation of MPO and interleukin (IL)-4, an increased infiltration of leukocytes in intestinal villi and increased expression of toll-like receptors (TLRs), such as TLR-2 and TLR-4 [10].

Several human studies focused on the possible relationship between the consumption of milk containing A1 or A2 beta-casein and milk intolerance. Different randomised crossover studies conducted on subjects with mild-to-moderate milk intolerance observed that A2-milk consumption could attenuate the acute gastrointestinal symptoms by reducing gastrointestinal transit time, lowered the inflammatory state that aggravated lactose intolerance symptoms, caused an increase of faecal SCFA content and improved cognitive performance [11,12,13,14,15].

The elderly population is increasing in Westernised countries and a growing scientific interest is focused on studying possible strategies to improve the quality of life and ameliorate health conditions of this particular category of people, thus reducing health care costs [16]. Ageing is characterised by some nutritional deficiencies, caused by several concurrent factors, such as appetite loss, impaired masticatory efficiency, reduced sensory perceptions, swallowing and digestion difficulties, delay in gastric emptying, decreased bowel motility, slower intestinal transit times and faecal constipation [17]. Ageing also affects the immune function, a process known as immunosenescence, and the intestinal microbiota composition, with a reduction in the numbers and diversity of many protective commensal anaerobes that play an important role in maintaining host health [18]. Within this context, milk consumption is particularly desirable for the elderly, as it contributes to the intake of important macro- and micro-nutrients, and research on the suitability of milk containing A2 beta-casein, characterised by a protein profile favouring a more physiological gastrointestinal transit in elderly subjects as compared to conventional milk, appears to be promising.

The aim of the present work was to analyse the effect of consumption of conventional milk, containing the A1/A2 beta-casein variant, compared to milk containing only the A2 beta-casein variant, in an animal model of ageing mice, to assess whether diet supplementation with bovine milk containing different beta-casein profiles could differently impact on gut health, by evaluating gut morphology and enzymatic activity, immunological phenotype, microbiota composition and short-chain fatty acids (SCFAs) production.

## 2. Materials and Methods

### 2.1. Milks

Milks containing A1 and A2 beta-casein (A1A2 milk) or only A2 beta-casein (A2A2 milk) were provided by Centrale del Latte d’Italia (Torino, Italy). The determination of the beta-casein isoform was attained by Real-Time PCR (Polymerase Chain Reaction) detection, on C > A substitution at position 473 of bovine beta-casein sequence, by a private laboratory of analysis, using an internally developed proprietary method. Milk samples were lyophilised, and freeze-stored until diets’ preparation. Compositional data of the two milk types were supplied by the provider and are shown in Supplementary Table S1.

### 2.2. Animals and Diets

Balb-c aged mice (20 months old) were kept at 23 °C with a 12 h light-dark cycle and fed ad libitum a standard laboratory pellet diet (4RF21, Mucedola, Milan, Italy). Mice had free access to food and water throughout the experiments. Animals were housed individually in stainless-steel cages and randomly divided into 3 groups (7–9 mice per group): the first group was fed a control diet (CTRL, *n* = 8), the second group received a diet containing A2A2 milk (A2A2, *n* = 9), while the third group received a diet containing A1A2 milk (A1A2, *n* = 7) for 4 weeks. Males and females were equally distributed among groups. Body weight and food intake were recorded every week and every other day respectively, throughout the experimental period. The control diet was a modified version of the AIN-93M maintenance diet for rodents, with an estimated total energy of 3.6 kcal/g [19], where casein was replaced with a casein-like amino acid mix (Table 1). All purified ingredients, except the lyophilised milks, were prepared and provided by Laboratori Dottori Piccioni s.r.l. (Gessate, Milan, Italy). Milk-supplemented diets were appropriately balanced for protein, fat and complex carbohydrate content (Table 1).

At the end of the experimental period, faeces were collected and stored at −80 °C for SCFAs profiling and microbiome analysis. Following overnight fasting, mice were anesthetised by intraperitoneal injection of pentobarbital (10 mg/kg), blood was drawn via cardiac puncture and serum was prepared and stored at −80 °C until further analysis. As protein-derived bioactive peptides mainly exert their action in the proximal part of the small intestine, specimens from duodenum and jejunum (sampled together) were excised, flushed with phosphate buffered saline (PBS) to remove all the content and placed in cold PBS for immediate flow cytometry analysis. Moreover, as in the present study experimental diets were administered for a longer period as compared to previous studies [9], distal portions of approximately 100 mg of jejunum and complete colon were quickly excised, flushed, immediately immersed in liquid nitrogen and later used for determination of enzymatic activities and of cytokine secretion respectively, in order to evaluate the caseins and protein-derived bioactive peptides’ effects on these organs. Small portions of spleen, liver, middle duodenum and ileum were washed, immediately placed in 10% buffered formalin solution and stored at room temperature for histological analyses (histomorphological and immunohistochemical evaluations). All experimental procedures involving animals complied with the European Guidelines for the Care and Use of Animals for Research Purposes (Directive 2010/63/EU) and protocols were approved by the Ethical Committee of the Food and Nutrition Research Centre and by the National Health Ministry, General Direction of Animal Health and Veterinary Drugs (agreement n° 388/2018-PR). All efforts were made to minimise suffering of the animals.

### 2.3. Lymphocytes Preparation from Mouse Small Intestine and Flow Cytometry Phenotypic Analysis

Intraepithelial lymphocytes (IELs) were isolated from the small intestine, as previously described [20], and resuspended at a concentration of 1 × 10^6^ cells/100 µL PBS for the staining. Peyer’s patches were removed from small intestine pieces before IELs isolation, to avoid accidental lymphocyte contamination from Peyer’s patches follicles. The monoclonal antibodies (eBioscience, Waltham, MA, USA) used are reported in Supplementary Table S2. After labelling, cells were centrifuged and resuspended in FACSFlow sheath fluid (BD Biosciences, Milan, Italy). Flow cytometry analysis was performed using a FACSCalibur flow cytometer (BD Biosciences). To exclude dead/dying cells and therefore nonspecific antibody binding cells, leukocytes were gated according to forward and side scatter parameters. The percentages of B lymphocytes and NK (Natural Killer) cells were calculated on leukocyte gate (CD45^+^), whereas the CD4^+^ and CD8^+^ subsets, as well as TCRγδ^+^ and TCRαβ^+^, were calculated on lymphocyte gate (CD3^+^). For all analyses, at least 10,000 events were acquired and analysed using the CellQuest software (BD Biosciences).

### 2.4. Cytokine Secretion in Intestinal Tissue

Colon was cut into small pieces and homogenised with pestles in glass tissue grinders with 1 mL PBS containing 1 mM phenylmethylsulphonylfluoride and Protease Inhibitor Cocktail (Complete Mini, Roche, Basel, Switzerland). Samples were subjected to two freeze–thaw cycles, followed by a sonication programme: 3 pulses of 30 s each, alternated with 2 stops of 15 s (Ultrasonic Liquid Processor S-4000 Misonix, Farmingdale, NY, USA), according to Reference [21]. After centrifugation at 15,000× *g* for 15 min at 4 °C, supernatants were recovered and stored at −80 °C until analysis. Protein concentration was determined by Bradford assay (SERVA Electrophoresis GmbH, Heidelberg, Germany). The levels of tumour necrosis factor (TNF)-α and IL-6 cytokines were determined by mouse ELISA (Enzyme Linked Immunoassorbent Assay) kits (Affymetrix, eBioscience, Waltham, MA, USA). Cytokine concentrations were expressed as per protein content of colonic tissue extracts (pg/mg).

### 2.5. Enzymatic Activity

Jejunum pieces were homogenised in 10 mM phosphate buffer pH 7, centrifuged at 20,000× *g* for 20 min at 4 °C. Supernatants were discarded, pellets homogenised again in 50 mM phosphate buffer pH 7 containing 0.5% hexadecyltrimethylammonium bromide (HETAB) and 10 mM Ethylenediaminetetraacetic acid (EDTA), immediately frozen in liquid nitrogen and then subjected to freeze–thaw and sonication cycles, as previously described [21].

An MPO (myeloperoxidase) enzymatic assay was performed according to Canali et al. [22]. For the DPP-4 (Dipeptidyl peptidase-4) enzymatic assay, a commercial kit was used (DPPIV/CD26 assay kit, Enzo Life Sciences Inc., Farmingdale, NY, USA), according to the manufacturer’s instructions. Enzymatic activities (optical density, OD/min) were expressed as per protein content of jejunal extracts, that were quantified by a 2DQuant kit following the manufacturer’s instructions (GE Healthcare Italia, Milan, Italy).

### 2.6. Histomorphological Evaluation

Samples of spleen, liver, middle duodenum and ileum were routinely embedded in paraffin wax blocks, sectioned at a thickness of 5 μm, mounted into glass slides and stained with haematoxylin and eosin (H&E) for histopathological examination. Each gut segment slide was captured with a Nikon DS-Fi1 digital camera coupled to a Zeiss AxioPhot microscope using a 20× objective lens. NIS-Elements F software was used for image capturing. Morphometric analysis was performed by Image^®^-Pro Plus software. The evaluated morphometric indices were: villus height (Vh, from the tip of the villus to the crypt), crypt depth (Cd, from the basis of the villus to the submucosa) and Vh/Cd ratio [23]. Morphometric analyses were performed on 10 well-oriented and intact villi and 10 crypts per sections of duodenum and ileum [24]. The observed histopathological findings were evaluated using a semiquantitative scoring system as follows: absent (score = 0), mild (score = 1), moderate (score = 2) and severe (score = 3), according to Sanches et al. [25]. The following histopathological alterations were evaluated: white pulp hyperplasia and depletion in spleen, vacuolar degeneration and lymphoplasmacytic infiltration in liver. Gut histopathological findings were separately assessed for mucosa (lymphoplasmacytic infiltrates) and submucosa (lymphoplasmacytic infiltrates and Gut-Associated Lymphoid Tissue (GALT) activation) for each segment. The gut scores resulted from the mean of the two gut segments scores (in turn obtained from addition of mucosa and submucosa scores). In order to investigate the accumulation of lipids and polysaccharides in the liver, frozen or formalin-fixed liver samples were also stained with Sudan Black and Periodic acid-Schiff (PAS), respectively. For PAS staining, formalin-fixed liver sections were brought to water, immersed in 0.5% periodic acid solution for 20 min, washed in running tap water for 5 min and immersed in Schiff’s reagent for a further 30 min. Sections were successively rinsed in running tap water for 10 min, dehydrated and mounted. For Sudan Black staining, frozen liver sections were brought to 70% ethanol for 1 min and immersed in the staining solution (Sudan III, Sudan IV, Herxheimer solution) for 1–2 min. Sections were rinsed in 50% ethanol for 1 min and then rinsed in water for 1–3 min, counterstained with Mayer haematoxylin, washed in running tap water and mounted. Lipid and polysaccharide staining intensities were semi-quantitatively scored as follows: grade 0 for absence of staining, grade 1 for mild staining, grade 2 for moderate staining and grade 3 for marked staining. All the slides were blind assessed by two independent observers and the discordant cases were reviewed, using a multi-head microscope, until unanimous consensus was reached.

### 2.7. Immunohistochemical Staining

Immunohistochemistry was performed on duodenum sections in order to characterise the inflammatory infiltrates. The characteristics of the employed antibodies and the corresponding working conditions are detailed in Supplementary Table S2. Immunohistochemical staining was performed by using the Vector Vectastain Elite ABC HRP kit (Vector Laboratories Inc., Burlingame, CA, USA). The sections were incubated with 0.3% hydrogen peroxide for 30 min at room temperature and then heated at 98 °C for 9 min in sodium citrate buffer or EDTA for antigen retrieval. Incubation with blocking serum lasted 20 min at room temperature. Slides were then incubated for 2 h with the primary antibodies in a humidified chamber at room temperature. Secondary detection was performed by using an avidin–biotin complex (ABC). The reaction was visualised by means of 3,3′-diaminobenzidine tetrahydrochloride (Sigma-Aldrich, St. Louis, MO, USA). Sections were counterstained with Mayer’s haematoxylin. Positive and negative immunohistochemistry controls were routinely used. Positive staining was evaluated by means of light microscopy. Five randomly selected high-power fields per each slide were captured with a Nikon DS-Fi1 digital camera coupled to a Zeiss AxioPhot microscope using a 20× objective lens, and NIS-Elements F software was used for image capturing and analysis. Quantification of immunohistochemical positivity (brownish cells) was then performed by Image^®^-Pro Plus software by means of pixel classification. The expression of CD8, CD45 and CD19 cell markers was estimated as the percentage of positive cells in the considered gut mucosal area (covering both the crypts and the villi).

### 2.8. Serum Immunoglobulins G (IgG) Measurements

IgG were quantified in serum with a mouse IgG ELISA Kit (Innovative Research Inc Peary Court, Novi, MI, USA), on 1:100,000 diluted samples, according to the manufacturer’s instructions. Data were expressed as mg/mL serum.

### 2.9. SCFA Analysis

Quantification of SCFAs was performed following the method of Canale et al. [26], with minor modifications. Frozen faecal samples (about 110 mg) were suspended in 1.1 mL 0.1 N H_2_SO_4_ solution and extensively vortexed. The mixture was centrifuged at 15,000× *g* for 10 min at 4 °C. The supernatant was transferred in a glass vial and 400 µL 0.1 N H_2_SO_4_ solution was added. Analyses were performed on a HPLC (High Performance Liquid Cromatography) Ultimate 3000 Thermo Fisher with autosampler equipped with a 300 × 7.8 mm Aminex HPX-87H and a guard-column. Injected samples (60 µL) were isocratically separated in 0.005 N H_2_SO_4_, at a flow rate of 0.6 mL/min and column temperature 41 °C. SCFAs were detected at 210 nm, using an external standard curve (0.9–3.6 lactic acid; 0.5–2 acetic and propionic acid; 0.47–1.88 butyric and isobutyric acid; 0.46–1.82 iso- and n-valeric acid) in 0.1 N H_2_SO_4_. Data were expressed as mg/g faecal fresh weight.

### 2.10. DNA Extraction from Faecal Samples and Next Generation Sequencing (NGS) Analysis

Total DNA was extracted from 80 mg faecal samples, as previously described [27]. The quality of isolated DNA was checked by NanoDrop spectrophotometer (Fisher Scientific, Schwerte, Germany). Partial 16S rRNA gene sequences were amplified using primer pair Pro341F: 5’-TCGTCGGCAGCGTCAGATGTGTATAAGAGACAGCCTACGGGNBGCASCAG-3’ and Pro805R: 5’-GTCTCGTGGGCTCGGAGATGTGTATAAGAGACAGGACTACNVGGGTATCTAATCC-3’, targeting the V3-V4 hypervariable region. Libraries were constructed following the standard protocol for MiSeq Reagent Kit V3 and sequenced on MiSeq platform (Illumina Inc., San Diego, CA, USA) at BMR Genomics S.r.l. sequencing laboratories (Padova, Italy). Raw reads are publicly available at the European Nucleotide Archive (ENA) under the accession number PRJEB37293.

### 2.11. Bioinformatics

Raw paired-end reads were processed using the R package DADA2 version 1.14.0. The Divisive Amplicon Denoising Algorithm (DADA), unlike the operational taxonomic units (OTU)s-based approach, that typically uses a 97% identity level to define taxonomic units, is based on the identification of single nucleotide sequence variants producing an amplicon sequence variant (ASV) table, which is a higher-resolution analogue of the traditional OTU table [28]. Reads containing ambiguous bases (Ns) were discarded, then the sequences were quality filtered by selecting the position where sequences showed a quality score drop as a trimming point (Phred < 30). The forward sequences were truncated to 290 bps and the reverse reads were truncated at 200 bps. The paired-end reads were merged (minimum overlap = 20 bps), the primers were trimmed, and the chimeras were excluded using the “consensus” option. The results of quality filtering steps are reported in Supplementary Table S3. Taxonomy was assigned with Silva database [29] version 132.

### 2.12. Statistical Analyses

Data from continuous variables were expressed as mean ± standard deviation (SD), while data from scores were expressed as median (interquartile range, IR). Variables were analysed by uni- and multi-variate approaches. Normal distribution and homogeneity of variance of biochemical, immunological and morphological parameters were assumed with Shapiro–Wilk’s and Levene’s tests, respectively. Statistical significance was evaluated by one-way analysis of variance (ANOVA), followed by post hoc Tukey’s honestly significant difference (HSD) test, or by the Kruskal–Wallis test, followed by post-hoc Dunn’s test, for normal and non-normal distributions, respectively. Differences with *p*-values < 0.05 were considered significant. Statistical analyses were performed with PAST software, version 3 [30], unless otherwise detailed. Intestinal morphometric indices were analysed by one-way ANOVA in the two different intestinal segments (duodenum and ileum) and by two-way ANOVA (IBM SPSS Statistics for Windows, Version 25.0; General Linear Model-Univariata), considering diet and intestinal segment as fixed factors.

Unsupervised principal component analysis (PCA) was performed with PAST software, considering colonic cytokines, small intestinal intraepithelial lymphocyte subpopulations, jejunal DPP-4, duodenal and ileal morphometric indices, duodenal immunohistochemical lymphocyte quantifications, serum IgGs and faecal SCFAs, as variables. Data were auto-scaled by mean-centring and normalised by SD on each variable. Missing data in the PCA were estimated by iterative imputation. A supervised Linear Discriminant Analysis (LDA) was run in PAST by including in the dataset all the variables that displayed a correlation factor above 0.6 absolute value on the 3 most representative principal components (PCs). The effect of the diet was tested on standardised values by PERMANOVA (Permutational Multivariate Analysis of Variance) (999 permutations) and by Bonferroni-corrected pairwise post hoc comparison.

The variability within bacterial communities (alpha diversity) was calculated as Shannon index in Phyloseq 1.30.0 R package [31] and the diet effect on alpha diversity was tested by ANOVA followed by Tukey’s test with *aov* and *TukeyHSD* functions. The variability among bacterial communities (beta diversity) was calculated as Bray–Curtis distance and the distance matrix was visualised with non-metric multidimensional scaling (NMDS). The effect of the diet on beta diversity was tested by PERMANOVA with *adonis* function with 999 permutations, the correlations between beta diversity and SCFA levels were tested with *envfit* function with 999 permutations and plotted as vectors on NMDS. All the analyses on beta diversity were conducted in the Vegan 2.5.6 R package [32]. The identification of the diet-related taxa was conducted on family-aggregated data using the LEfSe (Linear discriminant analysis Effect Size) procedure [33], with a threshold for the logarithmic linear discriminant analysis (LDA) score of 2.

## 3. Results

### 3.1. Milk Components and Lipid Profile

In order to verify the presence of the two different beta-casein allele variants, the C > A single nucleotide substitution at position 473 of bovine beta-casein sequence was evaluated by an external laboratory using Real-Time PCR in the two milk batches, confirming that A1A2 milk was heterozygous, while A2A2 milk was homozygous for the A2 variant. Chemical composition analysis showed limited differences between A1A2 and A2A2 milks for lactose, protein and fat content (around 0.1 g/100 mL). The lipid composition of the two milks showed minor differences in the relative percentage of most represented fatty acids (Supplementary Table S1).

### 3.2. Body Weight and Food Intake

No significant difference in food intake and body weight was observed among groups during the experimental period (Table 2).

### 3.3. Gut Immune Phenotyping and Cytokine Secretion

Flow cytometry analysis of the main leukocyte subpopulations in the jejunum and duodenum intraepithelial compartments revealed increased proportions in A2A2 mice of T helper CD4^+^ and B CD19^+^ lymphocytes (*p* ≤ 0.001 vs. CTRL and A1A2 for CD4^+^ and vs. CTRL for CD19^+^), accompanied by decreased pro-inflammatory T cytotoxic CD8^+^ T lymphocytes (*p* < 0.01 vs. CTRL and A1A2) (Table 3), indicating that A2A2 milk supplementation could significantly modify the gut immune phenotype of old mice as compared to A1A2 milk and CTRL diets. Both milk-supplemented diets induced a significant increase in NK cell percentage as compared to CTRL (*p* ≤ 0.001), whereas γδ T lymphocytes were increased in A1A2 mice, as compared to both CTRL and A2A2 (*p* < 0.01, Table 3). No significant differences among the three groups were observed in αβ T lymphocytes percentages, nor in pro-inflammatory IL-6 and TNF-α cytokine secretion in colon (Table 3).

### 3.4. Gut Enzymatic Activities

Both milk-supplemented diets displayed undetectable MPO activity in the jejunum, while MPO activity was detected in CTRL samples. No difference was observed in DPP-4 activity among the three groups (Table 3).

### 3.5. Histomorphological Evaluation

The effect of the different diets on the morphometric indices of aged mice is shown in Table 3. In the duodenum, Vh and Vh/Cd were significantly higher in A2A2 when compared to the A1A2 group, while Cd was higher in CTRL as compared to the A2A2 group (*p* < 0.05). In the ileum, Vh, Cd and Vh/Cd did not show any significant difference among the dietary treatments. Moreover, the two-way ANOVA showed that all the evaluated morphometric indices were influenced by diet (*p* < 0.05) and by intestinal segment (*p* < 0.001), while only Vh/Cd was also influenced by the interaction between the two factors (*p* < 0.05). Apart from diet, duodenum showed higher Vh, Cd and Vh/Cd than ileum, as expected from cranio-caudal physiological progression of intestinal morphometric indices (Table 3).

**Figure 1:**
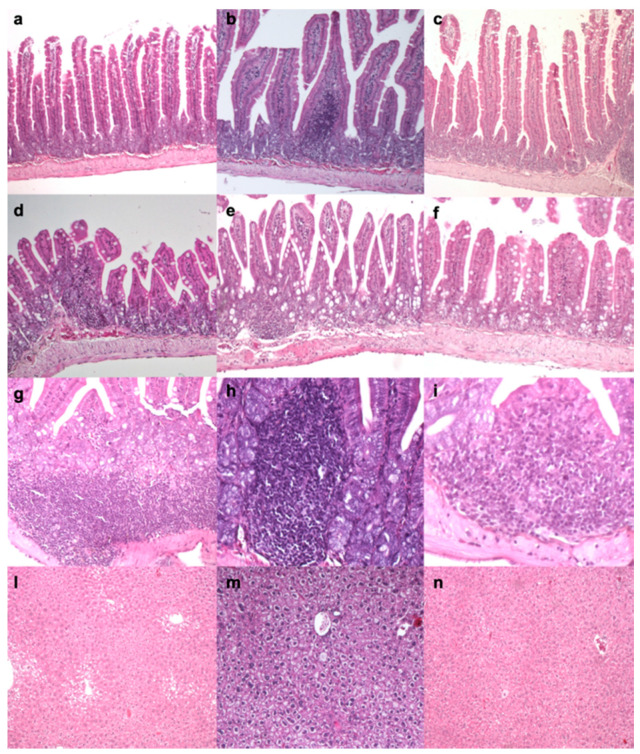
(**a**) Duodenum, A1A2 Mild and multifocal lymphoplasmacytic infiltration of the mucosa. Haematoxylin and eosin (H&E), 10×. (**b**) Duodenum, A2A2. Mild and multifocal lymphoplasmacytic infiltration of the mucosa. Haematoxylin and eosin (H&E), 10×. (**c**) Duodenum, CTRL. Mild and multifocal lymphoplasmacytic infiltration of the mucosa. Haematoxylin and eosin (H&E), 10×. (**d**) Ileum, A1A2. Mild and multifocal lymphoplasmacytic infiltration of the mucosa. H&E, 10×. (**e**) Ileum, A2A2. Mild and multifocal lymphoplasmacytic infiltration of the mucosa. H&E, 10×. (**f**) Ileum, CTRL. Mild and multifocal lymphoplasmacytic infiltration of the mucosa. H&E, 10×. (**g**) Duodenum, A1A2. Higher magnification of the lymphoplasmacytic infiltration in the mucosa. H&E, 20×. (**h**) Duodenum, A2A2. Higher magnification of the lymphoplasmacytic infiltration of the mucosa. H&E, 20×. (**i**) Duodenum, CTRL. Higher magnification of the lymphoplasmacytic infiltration of the mucosa. H&E, 20×. (**l**) Liver, A1A2. Moderate and diffuse vacuolar degeneration. H&E, 10×. (**m**) Liver, A2A2. Severe and diffuse vacuolar degeneration. H&E, 10×. (**n**) Liver, CTRL group. Mild and diffuse vacuolar degeneration. H&E, 10×.

In all groups, histopathological alterations varied from absent/mild to severe in gut and liver, while spleen showed no lesions. In particular, gut showed slightly higher lymphoplasmacytic infiltration in A1A2 (Figure 1a,d,g) and A2A2 groups (Figure 1b,e,h), as compared to CTRL (*p* < 0.002, Table 4, Figure 1c,f,i), but no significant differences were recorded between the two milk diets (A1A2 and A2A2). No significant differences were recorder for liver lymphoplasmacytic infiltration among groups (Table 4). Moreover, liver showed a higher degree of vacuolar degeneration in A1A2 (median score: 2.00, Figure 1l) and A2A2 (median score: 3.00, Figure 1m) groups, as compared to CTRL (median score: 1.00, *p* < 0.04, Table 4, Figure 1n). No polysaccharide accumulation was observed in hepatocytes (absence of PAS positivity), while mild to moderate multifocal steatosis was recorded by Sudan Black staining intensity in all groups, being slightly greater in A1A2 milk when compared to CTRL (Table 4).

### 3.6. Immunohistochemical Staining

The results for the immunohistochemical characterisation of lymphocytic infiltrates in duodenum are reported in Table 3. Multifocal to diffuse positivities were found in all the samples in all the groups, and the expression of CD8, CD19 and CD45 was not affected by dietary treatments (Supplementary Figure S1).

### 3.7. Analysis of Serum IgG

No significant differences in IgG serum levels were observed among the three groups (Table 3).

### 3.8. Analysis of Faecal SCFAs

Both SCFAs total contents and profiles were affected by the different diets: the supplementation of milks in murine diets significantly increased the faecal content of SCFAs, that doubled as compared to the CTRL group (*p* < 0.01, Table 3). The increase was specifically relevant for isobutyrate, whose content was 3-fold higher in both A1A2 and A2A2 groups, although only in the latter was the statistical significance reached, due to higher inter-individual variations in the first group.

### 3.9. Multivariate Profiling of the Effect of the Different Diets

**Figure 2:**
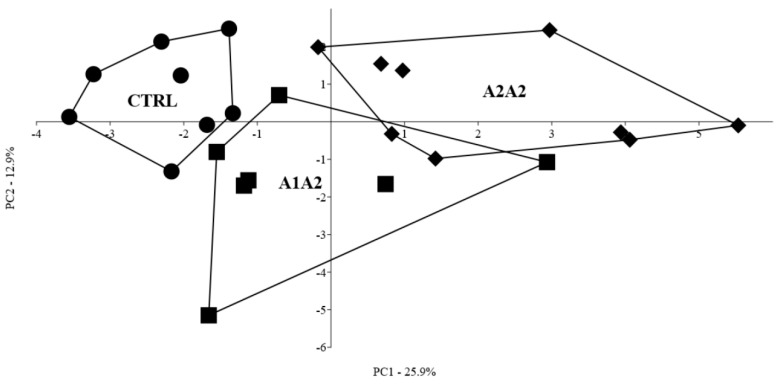
Principal component analysis (PCA) of intestinal immunological and morphological parameters, and faecal SCFAs measured on mice fed differently supplemented diets. CTRL, control diet (●); A1A2, milk-based diet with A1/A2 beta-casein variant (■); A2A2, milk-based diet with A2/A2 beta-casein variant (♦); PC, principal component. 8 CTRL, 7 A1A2 and 9 A2A2 mice were analysed.

Intestinal immunological and morphological parameters, gut enzymatic activities, serum IgGs and faecal SCFAs profiling were further explored by PCA. The first three PCs accounted for 50.2% of the overall explained variance, with individual values of 25.9%, 12.9% and 11.4% for PC1, PC2 and PC3, respectively. PCA correlation loadings on the first three PCs (accounting for more than 10% variability each) are shown in Supplementary Table S4. Although inter-individual variability was high, CTRL mice grouped closer on the first PC, as opposed to the A2A2 group, while the A1A2 group showed an intermediate positioning on the first PC. Some further distancing between CTRL and A2A2 groups, and the A1A2 group, could be observed along the second PC (Figure 2). The variables mostly contributing to such discrimination are identified by correlation loadings > 0.6 absolute value on the first three PCs (indicated in bold characters in Supplementary Table S4).

In order to maximise the differences among groups, a supervised Linear Discriminant Analysis (LDA) was conducted by including only the above-mentioned most discriminant variables. LDA correctly discriminated 66.7% of the individuals (Supplementary Figure S2). The CTRL and A1A2 groups were characterised by higher intestinal pro-inflammatory T cytotoxic CD8^+^ T lymphocytes and increased duodenal Cd, with respect to the A2A2 group. The latter was characterised by increased numbers of T helper CD4^+^ and B CD19^+^ lymphocytes and increased duodenal Vh/Cd. Both milk-supplemented groups, in particular those receiving A2A2 milk, showed higher NK percentages, as well as more total faecal SCFAs and isobutyrate. A1A2 milk was, furthermore, characterised by higher proportions of CD3 γδ cells and lower ileal Cd. PERMANOVA analysis confirmed that the three groups were significantly different (*p* < 0.001).

### 3.10. Analysis of Faecal Microbiota Composition

The effects of supplementation with different milks on faecal microbiota composition were evaluated by assessing the relative abundance of the main taxonomic bacterial groups by NGS. Quality checks returned a total of 1,485,831 reads that were clustered in 729 amplicon sequences variants (ASVs (, which were distributed among the samples, as reported in Supplementary Table S3.

The data analysed at amplicon sequences variants (ASVs) level revealed no difference for the dietary factor on alpha diversity (*p* = 0.42; Supplementary Figure S3a), while significant differences emerged by considering beta diversity (*p* = 0.001; CTRL vs. A1A2 = 0.0102; CTRL vs. A2A2 = 0.0002; A1A2 vs. A2A2 = 0.0339; Supplementary Figure S3b).

**Figure 3:**
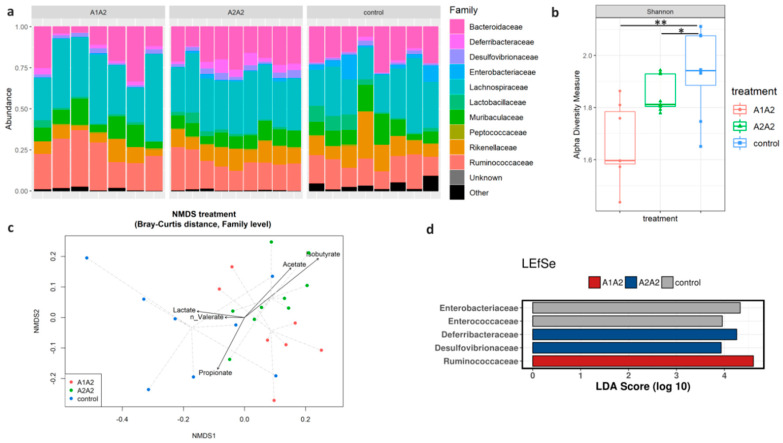
Family level analysis by Next Generation Sequencing (NGS) of faecal samples of mice fed differently supplemented diets. CTRL, control diet (*n* = 8); A1A2, milk-based diet with A1/A2 beta-casein variant (*n* = 7); A2A2, milk-based diet with A2/A2 beta-casein variant (*n* = 9). (**a**) Top 10 Families relative abundances. Each bar refers to a single sample. Colour-coding of bacterial families is shown on the right side. “Unknown” includes sequence variants not classified at the family level. “Other” includes the families other than the top 10 for relative abundances. (**b**) Shannon index boxplots. The lower and upper limits of the box correspond to the first and third quartiles, respectively. The horizontal line within the box represents the median. The vertical line extending from the top of the box indicates the maximum value, while the vertical line extending from the bottom of the box indicates the minimum value. The symbols represent the values for each individual sample. The asterisks indicate significant differences: * *p* < 0.05, ** *p* < 0.01. (**c**) Non-metric multidimensional scaling (NMDS) on Bray–Curtis distance matrix, each point is connected to the centroid group by ‘spider diagram’ (dashed lines). The results from *envfit* analysis are reported as vectors: the arrow shows an increasing gradient direction and the length of the vector is proportional to the correlation between variable and ordination. (**d**) LEfSe (Linear discriminant analysis Effect Size) results, the threshold applied for the logarithmic linear discriminant analysis (LDA) score was 2.

Taxonomic assignment revealed *Firmicutes* (mean = 55.5%; SD = 6.4%) and *Bacteroidetes* (mean = 37.7%; SD = 4.5%) as dominant phyla on overall samples, followed by *Proteobacteria* (mean = 4.3%; SD = 2.1%) and *Deferribacteres* (mean = 2.2%; SD = 1.7%) (Supplementary Figure S4). Moving through the taxonomic hierarchy at the genus level, 27% of total reads resulted to be unclassified (Supplementary Figure S5), while almost all the reads (99.9%) were classified at the family level (Figure 3a). For this reason, in order to lose as little information as possible, subsequent analyses were focused on family-aggregated data. At the family level, significant differences were reported for both alpha diversity (*p* = 0.002; CTRL vs. A1A2 = 0.002; A1A2 vs. A2A2 = 0.028; A2A2 vs. CTRL = 0.379, Figure 3b) and beta diversity (*p* = 0.006; CTRL vs. A1A2 = 0.035; A1A2 vs. A2A2 = 0.018; A2A2 vs. CTRL = 0.099, Figure 3c). The *envfit* analysis revealed a correlation between the increasing level of isobutyrate in the A2A2 group and the composition of bacterial community (*r*^2^ = 0.347, *p* = 0.030; Figure 3c). In order to identify bacterial taxa characterising each treatment group, LefSe analysis was applied to family-aggregated data, allowing the detection of *Deferribacteraceae* and *Desulfovibrionaceae* as the most discriminant families for the A2A2 group, while *Ruminococcaceae* were discriminant for the A1A2 group and *Enterobacteriaceae* and *Enterococcaceae* for the CTRL group (Figure 3d; Supplementary Figure S6).

## 4. Discussion

Recent evidence suggests that consumption of milk proteins in the elderly is effective for improving muscle strength, and also calcium and phosphorus are of great importance for this population group, as they play both structural and functional roles in bone and muscle, thereby reducing the risk of falls and fractures. However, in Italy, similarly to most European countries, the consumption of milk and dairy products has been progressively decreasing [34], due to both the consumers’ scepticism about health effects of dairy products [35] and to the increased digestive discomfort after dairy product consumption associated to lactose malabsorption, that affects about 65% of adults worldwide [11]. The involvement of dairy components other than lactose is becoming an increasingly suggestive hypothesis to explain milk intolerance [11]. The potential role of proteins, in particular A1 and A2 beta-casein variants, is gaining particular interest, as the digestion of the beta-casein variant A1 leads to the formation of BCM-7, a bioactive peptide associated with delayed milk gastrointestinal transit time. Despite this controversial role of BCM-7 on human health [8], so far, few studies have compared the impact of A1 and A2 beta-casein on gut health.

In the present work, we investigated the effect of milk containing different beta-casein profiles on the gut health of ageing mice, as compared to a control diet containing a casein-like amino acid mix. We hypothesised that the ageing mice physiology could represent a suitable model to mimic a chronic (sub)inflammatory status, that might emphasise the effects of the release of BCM-7 during digestion.

Despite the fact that no difference was observed in body weight and in food intake among the three groups, we found that the three diets differently affected the gut health status in the ageing mice population. The multivariate analysis of the parameters chosen for evaluating gut morphology and enzymatic activity, immunological phenotype and microbiota composition allowed to significantly cluster the animals according to the diet type. Although the dominant phyla on overall samples were *Firmicutes* and *Bacteroidetes*, followed by *Proteobacteria* and *Deferribacteres*, at the family level, the three dietary groups showed significant differences in microbial diversity, both in terms of richness and evenness (alpha diversity), and in composition (beta diversity). We observed a reduced alpha diversity at the family level in milk-supplemented mice with respect to the CTRL group, and this can appear quite surprising. However, such differences could be due to the distinct lipid content characterising milk-supplemented diets with respect to CTRL diets, since it is known that fat from diverse sources can exert different effects on gut microbiota [36]. Concerning discriminant families, *Enterobacteriaceae* and *Enterococcaceae* were scored in the CTRL group: these families were previously correlated with ageing and increased serum level of pro-inflammatory cytokines [18] and include several genera and species representing opportunistic pathogens, such as *Enterococcus faecium* and *Enterococcus faecalis*, involved in severe inflammatory and immune-mediated intestinal damages [37]. Moreover, a clusterisation was found in the gut microbiota of ageing mice fed both milk-supplemented diets, that were mainly characterised by enrichment in *Deferribacteraceae*, *Desulfovibrionaceae* and *Ruminococcaceae*. *Deferribacteraceae*, Gram-negative bacteria that produce energy by anaerobic respiration [38], are one of the predominant families in healthy mouse gut microbiome, displaying an upregulation of genes involved in cofactor and vitamin metabolism and in amino acid metabolism [39]. On the other hand, *Desulfovibrionaceae* include sulphate-reducing bacteria considered as endotoxin producers and may generate hydrogen sulphide that induces gut mucosal and hepatic injury [40]. This family has already been reported as food ingredient-susceptible indigenous bacteria in the gut of mice fed a diet containing 20% milk casein and 17% beef tallow [41], whose prevalence was directly correlated to the amount of dietary casein. *Ruminococcaceae* represents an important family of butyrate-producing bacteria. The presence of *Ruminococcaceae* in the intestine of mice receiving milk diets could be linked to the higher production of SCFAs observed in these groups. A2A2 milk-fed mice showed the highest faecal SCFAs and isobutyrate contents.

SCFAs are known to play a potential role in glucose homeostasis, lipid metabolism and body weight control, in regulating the immune system and inflammatory response [42]. It has been recently described that SCFAs produced by gut microbiota boost host antibody responses [43]. In particular, butyrate is effective in increasing the total number of CD4^+^ and decreasing a relative amount of CD8^+^ memory T cells in rat mesenteric lymph nodes through a mechanism not yet well understood [44]. Accordingly, in the A2A2 milk-supplemented group, we found a distribution of lymphocyte subpopulations in the intraepithelial compartment, characterised by significantly increased level of CD4^+^, CD19^+^ and NK, and a corresponding decrease of CD8^+^ cells, as compared to the other diets. These data suggest that A2A2 milk supplementation could counteract some of the ageing-associated immune alterations, that lead to increased susceptibility to infections, decreased vaccination response and to the onset of chronic inflammatory status, referred as inflammageing [45]. Indeed, the impairment of CD4^+^ T cell functions with ageing most certainly plays an important role in the reduced humoral response observed in older individuals. Through their B cell helper functions, CD4^+^ T cells play a major role in the immune response and it is now well recognised that CD4^+^ T cells are absolutely required for high-affinity antibody production by B cells [46]. NK cells are also found to be severely altered in the elderly, and they change their phenotype, showing a decreased expression of cytotoxicity activating receptors [47]. High levels of luminal SCFAs, besides the shaping of lymphocyte populations, have also been reported to increase the levels of systemic blood IgGs [43], thus exerting a significant role in regulating both mucosal and systemic antibody responses. In our study, we did not observe such effect on IgGs serum levels, suggesting that the improved IELs profile found by supplementing the ageing mice with A2A2 milk does not reflect a change in the systemic immune response and is rather localised, in particular in the proximal sections of the intestine. This hypothesis is supported by the histological findings at the intestinal level, that revealed an improved tropism of duodenal villi following A2A2 milk supplementation, as compared to A1A2 milk. This effect was not seen at the ileum level. Indeed, it is known that milk caseins are extensively digested in the stomach and in the proximal part of the small intestine, so that protein-derived bioactive peptides can hardly reach the distal part [48]. Consistently, BCM-7 and other related peptides were found in the jejunum after beta-casein consumption [49]. The observation that feeding A2A2 milk could improve the intestinal morphology, as well as improving the IELs profile, thus promoting a better absorption and functionality of the proximal intestine, can have very important implications for the elderly population, in which a reduction of absorption capacity due to intestinal wall thinning, as well as alteration and reduction of villi number, have been observed [50].

On the other hand, A1A2 milk-supplemented mice, as compared to CTRL and A2A2-fed mice, showed an increase of γδ^+^CD3^+^ T cells in IELs. The γδ^+^CD3^+^ T cells, particularly those expressed in the intraepithelial compartment, have been associated to epithelial barrier maintenance and repair [51]. This suggests that some mechanisms of repair could have been induced in the A1A2 milk-supplemented group, although the histological analysis did not indicate the onset of any significant differential inflammatory status. Duodenal immune-histochemical detection of leukocyte infiltration also revealed a similar degree of gut histopathological alterations and infiltrates, among the different dietary treatments, suggesting that these slight alterations could have been caused by the ageing process, rather than having a specific dietary origin. The apparent discrepancy between immunohistochemical data and flow cytometry analysis could be explained considering the different cellular compartments that had been evaluated.

Both milk supplementation types shared a common pattern as far as the intestinal inflammation status is concerned. Inflammation level was not affected by milk supplementation, neither at the distal intestinal level, as revealed by cytokine (TNF-alpha and IL-6) levels, nor in the jejunum, as shown by MPO activity determination. These results are in contrast with the findings of Ul Haq et al. [10], who observed a significant increase of MPO activity and other inflammatory markers in mouse intestinal tissue induced by A1 beta-casein supplementation. Probably, the discrepancy is due to differences in the experimental design, since in the present study, mice received a diet containing whole milk, while in Ul Haq et al.’s [10] study, the animals were supplemented with the purified beta-casein variants. Our results confirmed the absence of brush border membrane alterations and inflammation in the jejunum, as indicated by similar DPP-4 activity among the three groups, differently from what was previously reported [9]. DPP-4 is a dipeptidyl peptidase localised in the brush border membrane, able to catabolise BCM-7 [52]. Inflammation, inducing the flattening of mucosal brush border, can cause the impairment of DPP-4 [53]. It is important to mention that Barnett and co-workers [9] measured DPP-4 activity after few days of milk feeding (36 to 84 h—acute model), while a longer milk consumption (30 days, in our study—chronic model) may probably result in a physiological attenuation of such an induction mechanism, which was reported, in any case, to not be mediated by a µ opioid signalling pathway.

Some vacuolar degeneration, in association to a mild/moderate multifocal steatosis, was observed in the liver of milk-supplemented mice. The different origin of dietary fat between a control and milk-based diet is probably responsible for such mild liver degeneration. While the control diet contained exclusively plant origin lipids, the milk-based diets were characterised by a significant amount of animal fats, with a higher saturated fatty acid content [25]. As a confirmation, no difference in the liver histopathology was observed between the A1A2 and A1A2 groups.

## 5. Conclusions

The impact of A1 and A2 beta-casein on gut health, in particular SCFA content and intestinal microbiota, is still largely unexplored and, to the best of our knowledge, this is the first study addressing this aspect in an ageing model. We observed that supplementing the diet of ageing mice with bovine milk containing different beta-casein profiles increased the intestinal levels of SCFAs, through modulation of gut microbiota. This was particularly evident when A2/A2 beta-casein-containing milk was provided. In these animals, the increased level of SCFAs affected gut immunological phenotype, favouring CD4^+^ T cells’ differentiation and resulting in improved gut villi morphology. This scenario is particularly relevant if it is considered that A2A2 milk is supplemented in an ageing physiology model. In conclusion, the supplementation with bovine milk seems to partially counteract the ageing effect on the gut health, in particular at the proximal level. Thus, the consumption of the A2A2 milk type may be suggested as a suitable strategy to achieve positive gut health outcomes in the ageing population.

## Figures and Tables

**Table 1:** Diet compositions.

Ingredient	Control Diet ^1^	Milk-Supplemented Diets ^2^
(g/Kg)		
Maize starch	467.5	455.5
Amino acid mix	140	110
Lyophilised milk ^2^	-	120
Maltodextrin	155	155
Sucrose	100	55
Soya oil	40	7
Cellulose	50	50
Mineral mix	35	35
Vitamin mix	10	10
Choline chloride	2.5	2.5
tert-Butylhydroquinone	0.008	0.008

^1^ AIN-93M, modified by substitution of casein with casein-like amino acid mix; ^2^ 120 g lyophilised milk contained: 45 g carbohydrate (lactose), 30 g protein, 33 g fat.

**Table 2:** Body weight and food consumption from CTRL, A1A2 or A2A2 mice.

	CTRL	A1A2	A2A2	*p*
Body weight (g)				
Initial	29.1 ± 3.1	28.3 ± 4.1	28.4 ± 3.8	0.909
Final	30.0 ± 3.7	29.6 ± 4.7	31.3 ± 3.9	0.664
Food intake (g/day)	4.7 ± 0.4	4.6 ± 0.6	4.5 ± 0.4	0.526

CTRL, control diet (*n* = 8); A1A2, milk-based diet with A1/A2 beta-casein variant (*n* = 7); A2A2, milk-based diet with A2/A2 beta-casein variant (*n* = 9). Values represent means ± standard deviation (SD). *p*: significance according to one-way analysis of variance (ANOVA).

**Table 3:** Quantification of gut immune phenotyping, cytokine secretion, gut enzymatic activities, histomorphological evaluation, immunohistochemical staining, serum immunoglobulins and faecal short-chain fatty acids (SCFAs) of CTRL, A1A2 or A2A2 mice.

Analysis	Parameter	Localisation	CTRL	A1A2	A2A2	
			mean ± SD	mean ± SD	mean ± SD	*p*-Value
Gut immune phenotyping and cytokine secretion	CD3CD4	D/J IE	5.13 ± 2.32 ^a^	5.13 ± 0.74 ^a^	12.3 ± 4.52 ^b^	**0.001 ^§^**
	CD3CD8	D/J IE	79.9 ± 5.04 ^a^	77.0 ± 3.36 ^a^	66.2 ± 8.82 ^b^	**0.004 ^§^**
	CD3αβ	D/J IE	56.3 ± 14.5	42.9 ± 7.13	48.6 ± 9.07	0.075 ^$^
	CD3γδ	D/J IE	36.8 ± 11.3 ^a^	51.5 ± 8.49 ^b^	34.2 ± 7.15 ^a^	**0.003 ^$^**
	CD45CD19	D/J IE	4.95 ± 4.05 ^a^	11.6 ± 2.49 ^ab^	21.6 ± 8.33 ^b^	**0.001 ^§^**
	CD45NK	D/J IE	1.81 ± 0.53 ^a^	3.13 ± 0.81 ^b^	3.65 ± 1.17 ^b^	**0.001 ^$^**
	TNF-α	Colon	5.47 ± 2.85	4.71 ± 2.76	5.44 ± 5.03	0.648 ^§^
	IL-6	Colon	6.31 ± 4.36	5.79 ± 3.14	5.47 ± 3.53	0.833 ^§^
Gut enzymatic activities	DPP-4	Jejunum	326 ± 102	267 ± 83.5	292 ± 103	0.513 ^$^
	MPO	Jejunum	29.4 ± 18.0	n.d.	n.d.	
Histomorphological evaluation	Vh	Duodenum	10.0 ± 1.76 ^a^	8.33 ± 0.58 ^b^	9.57 ± 0.63 ^a^	**0.034 ^§^**
	Cd	Duodenum	1.18 ± 0.15 ^a^	1.09 ± 0.11 ^ab^	0.99 ± 0.14 ^b^	**0.030 ^$^**
	Vh/Cd	Duodenum	8.51 ± 1.20 ^ab^	7.72 ± 1.09 ^a^	9.86 ± 1.71 ^b^	**0.018 ^$^**
	Vh	Ileum	3.40 ± 0.42	3.21 ± 0.35	3.33 ± 0.31	0.578 ^$^
	Cd	Ileum	0.89 ± 0.09	0.79 ± 0.13	0.84 ± 0.08	0.166 ^$^
	Vh/Cd	Ileum	3.81 ± 0.39	4.13 ± 0.78	3.98 ± 0.51	0.558 ^$^
Immunohistochemical staining	CD8^+^ IHC	Duodenum	1.79 ± 0.79	2.07 ± 1.48	3.11 ± 1.47	0.082 ^§^
	CD19^+^ IHC	Duodenum	1.97 ± 0.77	1.72 ± 1.01	1.45 ± 0.69	0.437 ^$^
	CD45^+^ IHC	Duodenum	3.05 ± 1.12	3.20 ± 1.11	4.11 ± 0.53	0.059 ^$^
Immunoglobulin	IgGs	Serum	2.72 ± 1.01	2.24 ± 1.29	2.65 ± 0.41	0.571 ^$^
Short-Chain Fatty Acids	Acetate	Faecal	6.70 ± 6.76	9.85 ± 13.6	14.8 ± 9.14	0.202 ^§^
	isobutyrate	Faecal	25.1 ± 10.5 ^a^	77.4 ± 62.0 ^ab^	80.4 ± 32.6 ^b^	**0.012 ^§^**
	n-valerate	Faecal	30.4 ± 17.4	39.8 ± 10.5	35.7 ± 8.09	0.618 ^§^
	SCFAs	Faecal	60.1 ± 20.9 ^a^	127 ± 79.2 ^b^	131 ± 42.6 ^b^	**0.008 ^§^**

Leukocyte subpopulations (% CD3^+^ for CD4^+^, CD8^+^, αβ, γδ, or % CD45^+^ for CD19^+^ and Natural Killer (NK), respectively), cytokines (pg/mg of protein), activity on dipeptidylpeptidase (DPP)-4 and myeloperoxidase (MPO, optical density (OD)/min/mg of protein), morphometric measurements (Vh: villus height; Cd: crypt depth; in mm); immunohistochemical (IHC) quantification of CD8^+^, CD19^+^, CD45^+^ (% positive cells); serum Immunoglobulin G (mg/mL); short-chain fatty acids (SCFAs, mg/g faecal fresh weight). CTRL, control diet (*n* = 8); A1A2, milk-based diet with A1/A2 beta-casein variant (*n* = 7); A2A2, milk-based diet with A2/A2 beta-casein variant (*n* = 9). SD: standard deviation. ^$^ *p*-value of analysis of variance by Fisher’s and Tukey’s post hoc test. ^§^ *p*-value of analysis of variance by Kruskal–Wallis and Dunn’s post hoc test. ^a,b^ Post hoc classes of uniformity when significance at *p* < 0.05 was assessed by one-way ANOVA. To facilitate reading, *p*-values < 0.05 are shown in bold.

**Table 4:** Effect of dietary treatments on the histopathological scores of CTRL, A1A2 or A2A2 mice.

Organ	Diet			*p*
	CTRL	A1A2	A2A2	
Gut, median (IR)	0.50 (0.0–1.0) ^a^	1.00 (1.0–2.0) ^b^	1.00 (1.0–2.0) ^b^	0.002
Spleen	Absence of alterations			
Liver, vacuolar degeneration (H&E), median (IR)	1.00 (0.0–2.7) ^a^	2.00 (1.0–4.0) ^ab^	3.0 (2.5–3.5) ^b^	0.04
Liver, lymphoplasmacytic infiltration, median (IR)	0.5 (0.0–1.0)	0.0 (0.0–1.0)	0.0 (0.0–1.0)	0.63
Liver, steatosis (Sudan Black), median (IR)	0.00 (0.0–1.0)	0.00 (0.0–2.0)	0.0 (0.0–1.00)	0.729

CTRL, control diet (*n* = 8); A1A2, milk-based diet with A1/A2 beta-casein variant (*n* = 7); A2A2, milk-based diet with A2/A2 beta-casein variant (*n* = 9). Data were analysed by Kruskal–Wallis and Dunn’s post hoc test. Medians labelled with different superscript letters (^a,b^) significantly differ (*p* < 0.05). IR = interquartile range.

## Supplementary Materials

The following are available online at https://www.mdpi.com/2072-6643/12/7/2147/s1: Table S1: Composition of A1A2 and A2A2 milk for lactose, protein and fat (g/100 mL), and their lipid profile (g/100 g fat), Table S2: List of antibodies and working conditions used for immunological profile analyses, Table S3: Reads’ quality checks, amplicon sequence variants and alpha diversity of faecal samples from A1A2, A2A2 and CTRL mice, Table S4: Principal component analysis (PCA) correlation loadings on the first 3 PCs, Figure S1: Representative images of immunohistochemistry performed on duodenum of CTRL, A1A2 and A2A2 mice. (**a**) Duodenum, group CTRL. Moderate and multifocal CD8 positivity. 20×, (**b**) Duodenum, group CTRL. Moderate and multifocal CD19 positivity. 20×, (**c**) Duodenum, group CTRL. Moderate and multifocal CD45 positivity. 20×, (**d**) Duodenum, group A1A2. Moderate and multifocal CD8 positivity. 20×, (**e**) Duodenum, group A1A2. Moderate and multifocal CD19 positivity. 20×, (**f**) Duodenum, group A1A2. Moderate and multifocal CD45 positivity. 20×, (**g**) Duodenum, group A2A2. Moderate and multifocal CD8 positivity. 20×, (**h**) Duodenum, group A2A2. Moderate and multifocal CD19 positivity. 20×, (**i**) Duodenum, group A2A2. Moderate and multifocal CD45 positivity. 20×, Figure S2: Linear Discriminant Analysis (LDA) and biplots of intestinal immunological and morphological parameters, and faecal SCFAs showing a correlation loading > 0.6 absolute value on the first 3 PCs, from mice fed differently supplemented diets. CTRL, control diet (●); A1A2, milk-based diet with A1/A2 beta-casein variant (■); A2A2, milk-based diet with A2/A2 beta-casein variant (**♦**). Cd-Duo and Cd-Ile: Crypth depth in duodenum and ileum; Vh/Cd Duo: Villus height/Crypth depth ratio in duodenum. TNFa: Tumour Necrosis Factor α, Figure S3: Alfa and beta diversity at Amplicon Sequences Variants (ASVs) level of faecal samples from A1A2, A2A2 and CTRL mice. (**a**) Shannon index boxplots. The lower and upper limits of the box correspond to the first and third quartiles, respectively. The horizontal line within the box represents the median. The vertical line extending from the top of the box indicates the maximum value, while the vertical line extending from the bottom of the box indicates the minimum value. The symbols represent the values for each individual sample. (**b**) Non-metric multidimensional scaling (NMDS) on Bray–Curtis distance matrix, each point is connected to the centroid group by ‘spider diagram’ (dashed lines), Figure S4: Top 5 Phyla relative abundances in faecal samples from A1A2, A2A2 and CTRL mice. Each bar refers to a single sample. Colour-coding of bacterial phyla is shown on the right side. “Unknown” includes sequence variants not classified at Phylum level. “Other” includes the Phyla other than the top 5 for relative abundances, Figure S5. Top 10 Genera relative abundances in faecal samples from A1A2, A2A2 and CTRL mice. Each bar refers to a single sample. Colour-coding of bacterial genera is shown on the right side. “Unknown” includes sequence variants not classified at Genus level. “Other” includes the Genera other than the top 10 for relative abundances, Figure S6: Boxplots of isobutyrate levels (mg/g faecal fresh weight) and of *Ruminococcaceae*, *Enterococcaceae*, *Enterobacteriaceae*, *Deferribacteriaceae* and *Desulfovibrionaceae* relative abundances in faecal samples from A1A2, A2A2 and CTRL mice. The lower and upper limits of the box correspond to the first and third quartiles, respectively. The horizontal line within the box represents the median. The vertical line extending from the top of the box indicates the maximum value, while the vertical line extending from the bottom of the box indicates the minimum value. The dots outside the boxplot represent the outliers.
