# Supplementary material for: A Comprehensive Evaluation of the Impact of Bovine Milk Containing Different Beta-Casein Profiles on Gut Health of Ageing Mice
Source: Nutrients. 2020 Jul 19;12(7):2147. doi: 10.3390/nu12072147 (PMC7400800; doi:10.3390/nu12072147)
Supplement: Supplementary file 1 [file nutrients-12-02147-s001.pdf]

Table S1. Composition of A1A2 and A2A2 milk for lactose, protein and fat (g/100 mL), and their lipid profile (g/100 g fat).

(g/100 mL)		A1A2	A2A2
Lactose		4.80	4.92
Protein		3.49	3.43
Fat		4.02	3.91
(g/100 g fat)			
Butyric	C4:0	2.2	2.2
Caproic	C6:0	1.8	1.9
Caprylic	C8:0	1.3	1.4
Capric	C10:0	3.4	3.8
Undecanoic	C11:0	0.14	0.36
Lauric	C12:0	4.8	5.1
Tridecanoic	C13:0	0.18	0.2
Myristic	C14:0	9.3	10.4
Myristoleic	C14:1	1.3	1.3
Pentadecanoic	C15:0	1.7	1.8
Palmitic	C16:0	32.3	34.3
Palmitoleic	C16:1	2	1.9
Margaric	C17:0	0.68	0.65
Heptadecenoic	C17:1	0.29	0.3
Stearic	C18:0	8	7.9
Oleic	C18:1	20.4	20.2
Linoleic n6	C18:2	8.4	5.1
All-trans-9,12,15-octadecatrienoic	C18:3	0.2	0.15
γ-Linolenic n6	C18:3	-	0.1
α-Linolenic n3	C18:3	0.39	0.37
Rumenic	C18:2	0.27	0.28
Behenic	C22:0	0.11	-
Erucic	C22:1	0.2	-
Eicosatrienoic n3	C20:3	-	0.19

Table S2. List of antibodies and working conditions used for immunological profile analyses.

Application	Antibody	Clone	Manufacturer	Antigen retrieval	Dilution
FC	FITC-CD3	500A2	eBioscience	-	0.5 μg/100 μL
	PE-Cy5-CD3	145-2C11	eBioscience	-	0.25 μg/100 μL
	FITC-CD4	RM4-5	eBioscience	-	0.25 μg/100 μL
	PE-CD8	53–6.7	eBioscience	-	0.25 μg/100 μL
	PE-CD19	1D3	eBioscience	-	0.25 μg/100 μL
	PE-pan NK	DX5	eBioscience	-	0.5 μg/100 μL
	PE-TCR γδ	GL3	eBioscience	-	0.5 μg/100 μL
	PE-Cy5-TCR αβ	H57-597	eBioscience	-	0.25 μg/100 μL
	PerCP-Cy5.5-CD45	30-F11	eBioscience	-	0.125 μg/100 μL
	CD16/CD32	93	eBioscience	-	0.5 μg/100 μL
IHC	CD8	polyclonal	Biorbyt	9' EDTA buffer, pH 8.0	1:800
	CD19	polyclonal	Elabscience	9' citrate buffer, pH 6.0	1:800
	CD45	polyclonal	Elabscience	9' EDTA buffer, pH 8.0	1:300

Abbreviations used: FC: flow cytometry, IHC: immunohistochemistry; FITC: fluorescein isothiocyanate, PE-Cy5: phycoerythrin-cyanin 5, PE: phycoerythrin, PerCP-Cy5.5: peridinin-chlorophyll-protein-cyanin 5.5.

Table S3. Reads quality checks, amplicon sequence variants and alpha diversity of faecal samples from A1A2, A2A2 and CTRL mice.

ID_BMR	ID_CREA	treatment	Input[a]	Filtered[b]	denoisedF[c]	denoisedR[d]	Merged[e]	Nonchim[f]	ASVs[g]	Shannon[h]
299368F280180	AC_1	CTRL	86,741	61,934	60,581	61,561	58,751	55,191	198	3.629

299369F280181	AC_2	CTRL	103,510	75,184	73,864	74,801	71,736	66,605	201	3.765
299370F280182	AC_3	CTRL	103,626	74,949	73,209	74,352	69,959	62,258	194	3.623
299371F280183	AC_4	CTRL	71,583	49,784	48,046	49,401	45,724	41,261	171	3.873
299372F280184	AC_5	CTRL	140,899	101,815	100,806	101,410	98,943	93,942	194	3.592
299373F280185	AC_6	CTRL	126,926	90,275	89,399	89,983	88,066	86,509	198	3.901
299374F280186	AC_7	CTRL	93,848	65,163	64,349	64,929	62,846	60,425	178	3.645
299375F280187	AC_8	CTRL	89,813	61,966	60,918	61,697	59,605	57,769	180	3.687
299376F280188	AS_9	A1A2 milk	89,575	64,434	63,287	64,078	61,832	59,449	188	3.626
299377F280189	AS_10	A1A2 milk	108,579	77,579	76,438	77,093	73,965	68,208	183	3.902
299378F280190	AS_11	A1A2 milk	123,359	89,534	88,342	89,088	85,733	81,727	224	4.330
299379F280191	AS_13	A1A2 milk	65,581	45,592	44,940	45,449	44,123	43,522	199	3.972
299380F280192	AS_15	A1A2 milk	120,206	86,923	85,880	86,514	83,960	80,800	206	3.869
299381F280193	AS_16	A1A2 milk	110,704	79,806	78,691	79,481	76,863	72,960	207	3.532
299382F280194	AS_17	A1A2 milk	95,218	67,229	66,413	67,023	65,290	62,531	183	3.646
299383F280195	AT_18	A2A2 milk	122,357	87,406	86,494	87,091	85,318	82,848	197	3.697
299384F280196	AT_19	A2A2 milk	73,599	52,530	51,540	52,262	49,869	48,189	212	4.037
299385F280197	AT_20	A2A2 milk	86,571	62,933	60,996	62,407	57,510	50,197	219	3.929
299386F280198	AT_21	A2A2 milk	101,387	74,439	72,332	73,836	68,509	57,014	184	3.766
299387F280199	AT_22	A2A2 milk	55,075	39,006	37,507	38,610	35,000	27,934	147	3.565
299388F280200	AT_23	A2A2 milk	109,282	78,705	76,405	78,070	71,961	58,740	210	3.964
299389F280201	AT_24	A2A2 milk	112,187	80,312	78,249	79,737	74,236	60,204	207	3.849
299390F280202	AT_25	A2A2 milk	99,292	71,861	70,046	71,109	66,827	58,076	178	3.689
299391F280203	AT_26	A2A2 milk	94,859	67,850	65,814	67,340	61,791	49,472	161	3.708

a: amount of raw reads; **b**: amount of reads after Ns filtering; **c**: amount of forward reads after Q30 denoising; **d**: amount of reverse reads after Q30 denoising; **e**: amount of reads after paired end merginf (minimun overlap cutoff= 20bps); **f**: amount of reads after chimera check; **g**: amount of Amplicon Sequence Variants; **h**: Shannon index values

Table S4. Principal component analysis (PCA) correlation loadings on the first 3 PCs.

Parameter	Localisation	PC 1 (25.9%)	PC 2 (12.9%)	PC 3 (11.4%)
**Acetate**	Faecal	**0.806**	0.090	-0.059
**CD3αβ**	Duod/Jejun	-0.261	0.446	0.527
**CD3CD4**	Duod/Jejun	**0.705**	0.229	-0.001
**CD3CD8**	Duod/Jejun	**-0.681**	-0.309	-0.099
**CD3γδ**	Duod/Jejun	-0.101	**-0.814**	-0.335

**CD19**	Duodenum	-0.086	-0.317	0.505
**CD45**	Duodenum	0.447	-0.218	0.516
**CD45CD19**	Duod/Jejun	**0.868**	0.066	0.123
**CD45NK**	Duod/Jejun	**0.651**	-0.027	-0.029
**CD8**	Duodenum	0.421	0.223	0.396
**Cd**	Duodenum	**-0.603**	-0.143	-0.139
**Cd**	Ileum	-0.168	**0.743**	-0.214
**DPP-4**	Jejunum	-0.129	0.592	-0.282
**IgG**	Serum	0.214	0.362	-0.362
**IL-6**	Colon	-0.054	0.061	0.570
**n-butyrate**	Faecal	**0.854**	-0.287	0.011
**n-valerate**	Faecal	0.513	-0.203	**-0.608**
**SCFAs**	Faecal	**0.886**	-0.246	-0.116
**TNF-α**	Colon	0.113	0.140	**0.631**
**Vh/Cd**	Duodenum	**0.630**	0.209	-0.111
**Vh/Cd**	Ileum	-0.026	-0.583	0.134
**Vh**	Duodenum	0.112	0.184	-0.278
**Vh**	Ileum	-0.203	0.241	-0.165

Positive or negative values indicate a direct or inverse correlation between variables and PCs, respectively. In bold values > ± 0.6 are shown. Abbreviations used: Cd: crypt depth, DPP: dipeptidyl peptidase, IL: interleukin, NK: natural killer, SCFAs: short chain fatty acids, TNF: tumor necrosis factor, Vh: villus height.

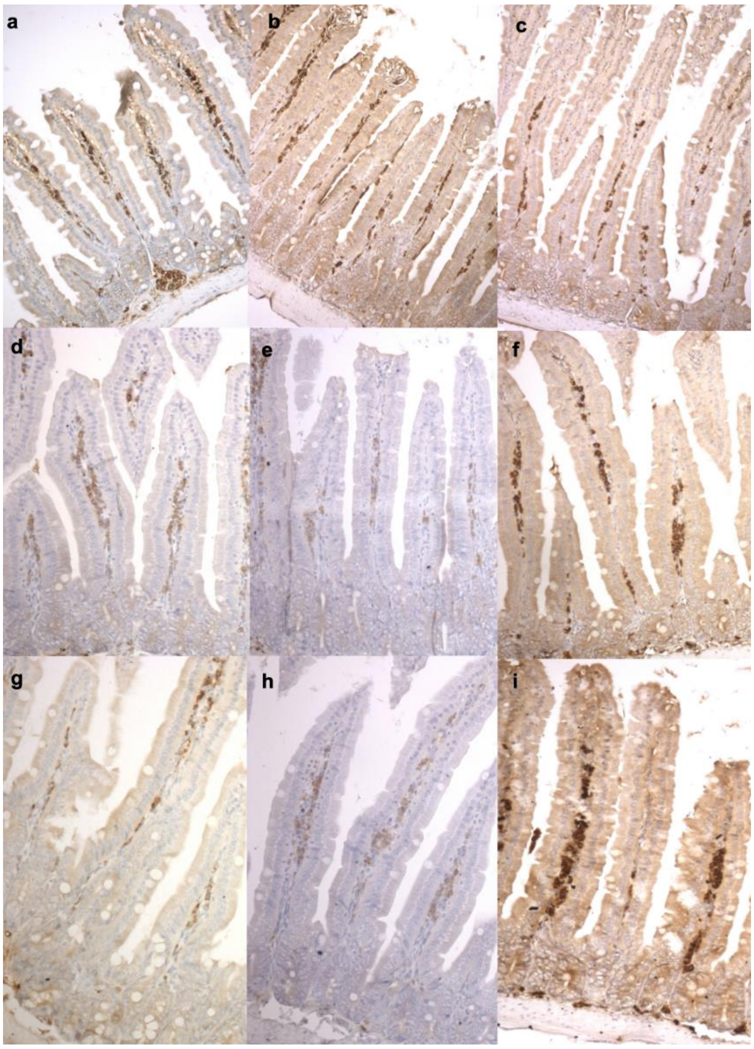

Figure S1. Representative images of immunohistochemistry performed on duodenum of CTRL, A1A2 and A2A2 mice. (**a**) Duodenum, group CTRL. Moderate and multifocal CD8 positivity. 20×, (**b**) Duodenum, group CTRL. Moderate and multifocal CD19 positivity. 20×, (**c**) Duodenum, group CTRL. Moderate and multifocal CD45 positivity. 20×, (**d**) Duodenum, group A1A2. Moderate and multifocal

CD8 positivity. 20×, (**e**) Duodenum, group A1A2. Moderate and multifocal CD19 positivity. 20×, (**f**) Duodenum, group A1A2. Moderate and multifocal CD45 positivity. 20×, (**g**) Duodenum, group A2A2. Moderate and multifocal CD8 positivity. 20×, (**h**) Duodenum, group A2A2. Moderate and multifocal CD19 positivity. 20×, (**i**) Duodenum, group A2A2. Moderate and multifocal CD45 positivity. 20×.

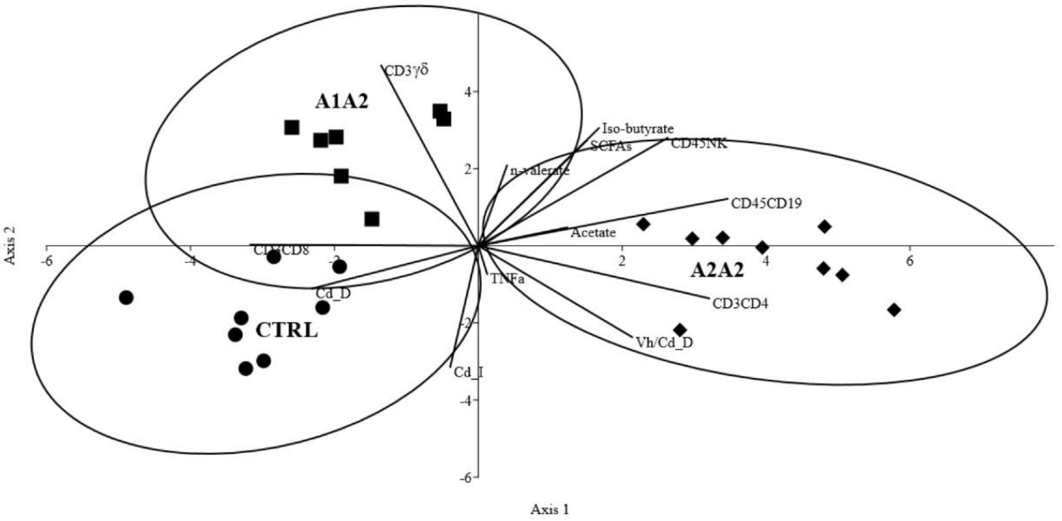

Figure S2. Linear Discriminant Analysis (LDA) and biplots of intestinal immunological and morphological parameters, and faecal SCFAs showing a correlation loading > 0.6 absolute value on the first 3 PCs, from mice fed differently supplemented diets. CTRL, control diet (●); A1A2, milk-based diet with A1/A2 beta-casein variant (■); A2A2, milk-based diet with A2/A2 beta-casein variant (♦). Cd-Duo and Cd-Ile: Crypth depth in duodenum and ileum; Vh/Cd Duo: Villus height/Crypth depth ratio in duodenum. TNFa: Tumour Necrosis Factorα.

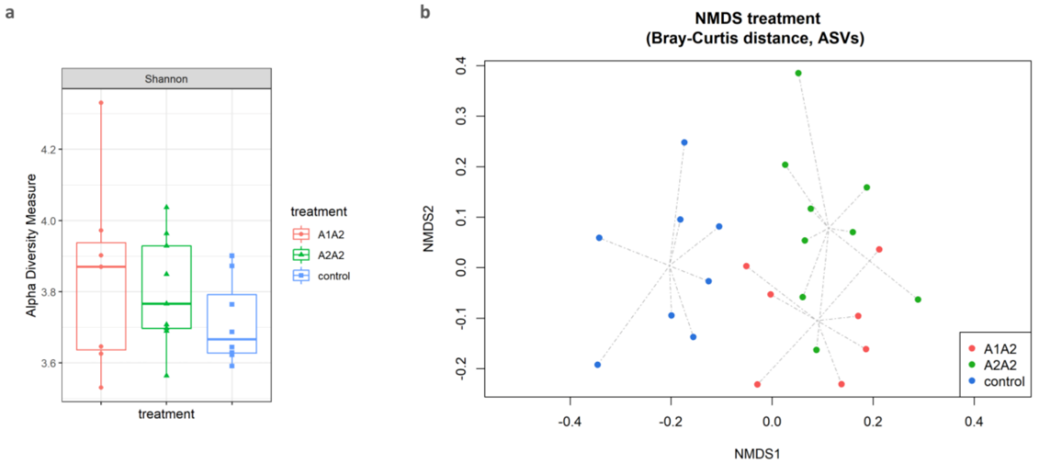

Figure S3. Alfa and beta diversity at Amplicon Sequences Variants (ASVs) level of faecal samples from A1A2, A2A2 and CTRL mice. (**a**) Shannon index boxplots. The lower and upper limits of the box correspond to the first and third quartiles, respectively. The horizontal line within the box represents the median. The vertical line extending from the top of the box indicates the maximum value, while the vertical line extending from the bottom of the box indicates the minimum value. The symbols represent the values for each individual sample. (**b**) Non-metric multidimensional scaling (NMDS) on Bray–Curtis distance matrix, each point is connected to the centroid group by 'spider diagram' (dashed lines).

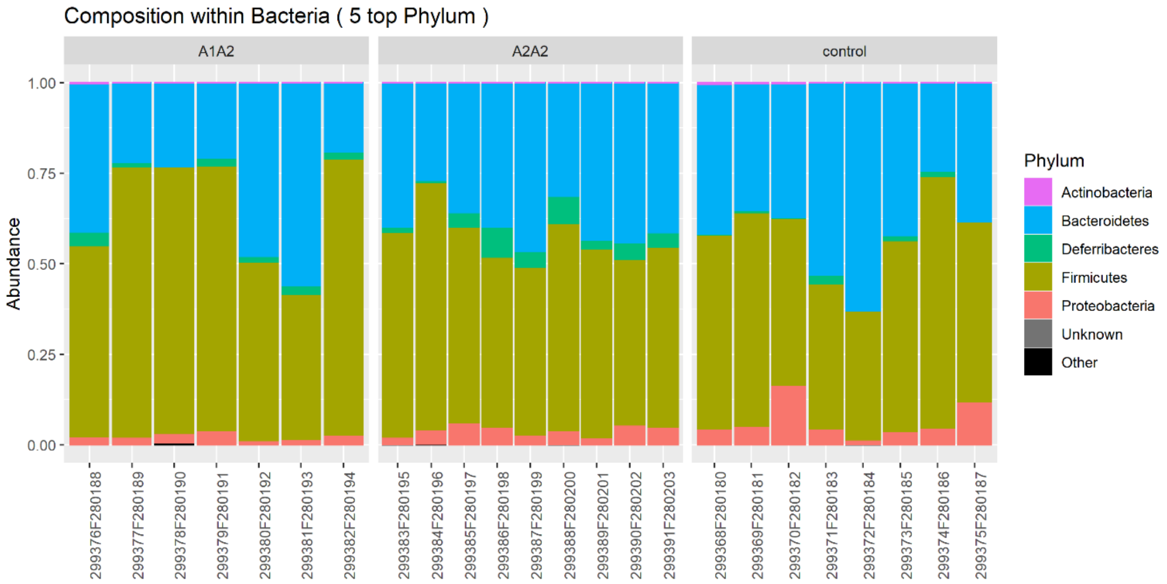

Figure S4. Top 5 Phyla relative abundances in faecal samples from A1A2, A2A2 and CTRL mice. Each bar refers to a single sample. Colour-coding of bacterial phyla is shown on the right side. “Unknown” includes sequence variants not classified at Phylum level. “Other” includes the Phyla other than the top 5 for relative abundances.

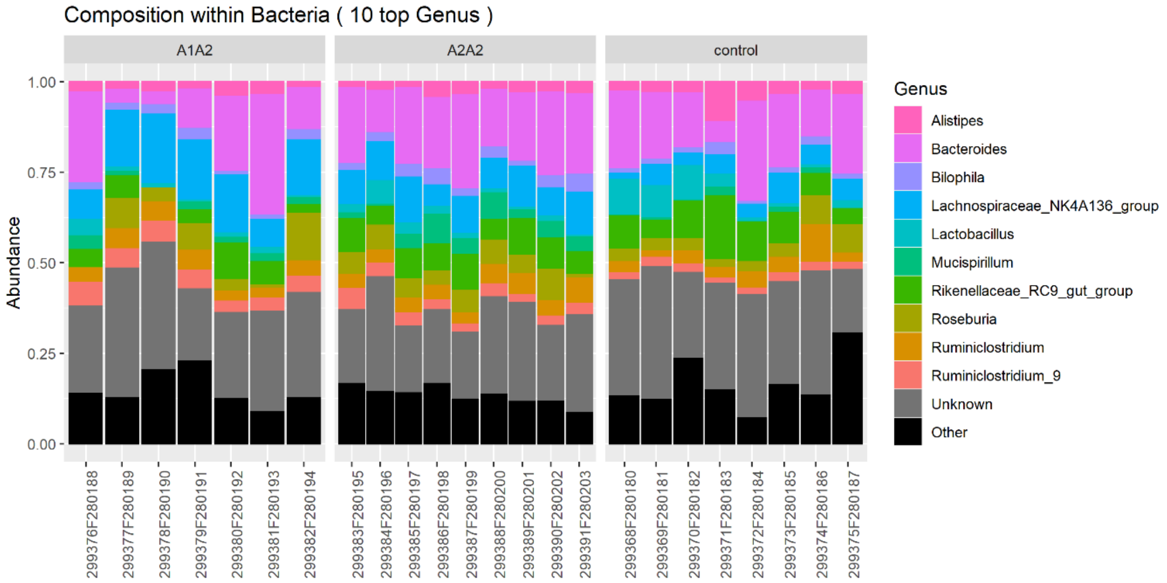

Figure S5. Top 10 Genera relative abundances in faecal samples from A1A2, A2A2 and CTRL mice. Each bar refers to a single sample. Colour-coding of bacterial genera is shown on the right side. “Unknown” includes sequence variants not classified at Genus level. “Other” includes the Genera other than the top 10 for relative abundances.

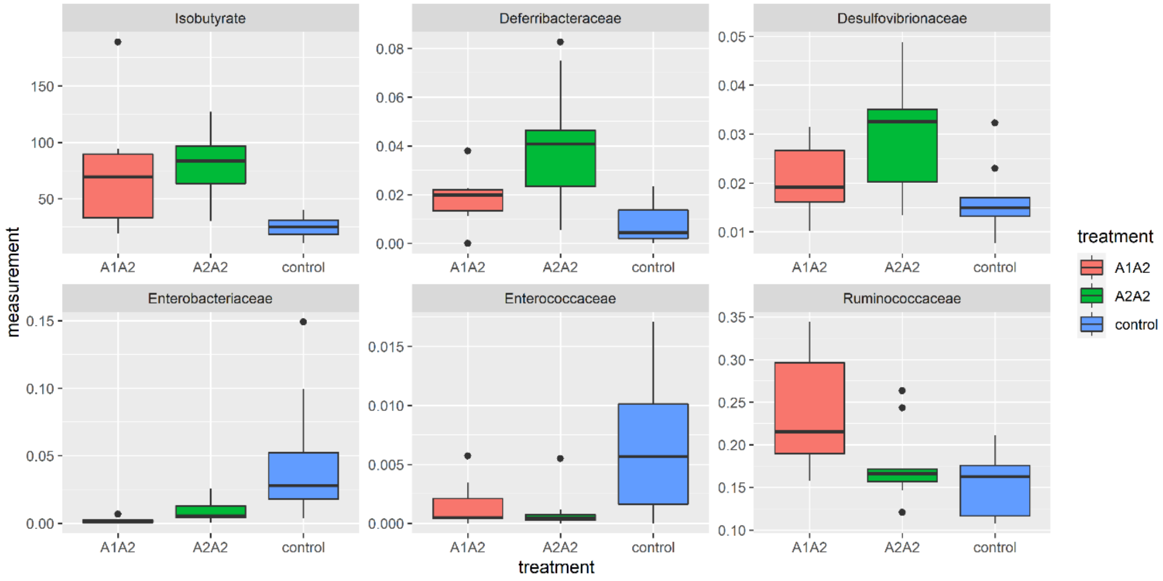

Figure S6. Boxplots of isobutyrate levels (mg/g faecal fresh weight) and of *Ruminococcaceae*, *Enterococcaceae*, *Enterobacteriaceae*, *Deferribacteriaceae* and *Desulfovibrionaceae* relative abundances in faecal samples from A1A2, A2A2 and CTRL mice. The lower and upper limits of the box correspond to the first and third quartiles, respectively. The horizontal line within the box represents the median. The vertical line extending from the top of the box indicates the maximum value, while the vertical line extending from the bottom of the box indicates the minimum value. The dots outside the boxplot represent the outliers.
